# Genotype × Environment Studies on Resistance to Late Leaf Spot and Rust in Genomic Selection Training Population of Peanut (*Arachis hypogaea* L.)

**DOI:** 10.3389/fpls.2019.01338

**Published:** 2019-12-04

**Authors:** Sunil Chaudhari, Dhirendra Khare, Sudam C. Patil, Subramaniam Sundravadana, Murali T. Variath, Hari K. Sudini, Surendra S. Manohar, Ramesh S. Bhat, Janila Pasupuleti

**Affiliations:** ^1^Crop Improvement- Asia Program, International Crops Research Institute for the Semi-Arid Tropics (ICRISAT), Hyderabad, India; ^2^Department of Plant Breeding and Genetics, Jawaharlal Nehru Krishi Vishwa Vidyalaya (JNKVV), Jabalpur, India; ^3^Oilseeds Research Station, Mahatma Phule Krishi Vidyapeeth (MPKV), Jalgaon, India; ^4^Coconut Research Station, Tamil Nadu Agricultural University (TNAU), Coimbatore, India; ^5^Department of Biotechnology, University of Agricultural Sciences, Dharwad, India

**Keywords:** G x E, GGE, genomic selection, peanut, training population

## Abstract

Foliar fungal diseases especially late leaf spot (LLS) and rust are the important production constraints across the peanut growing regions of the world. A set of 340 diverse peanut genotypes that includes accessions from gene bank of International Crops Research Institute for the Semi-Arid Tropics (ICRISAT), elite breeding lines from the breeding program, and popular cultivars were screened for LLS and rust resistance and yield traits across three locations in India under natural and artificial disease epiphytotic conditions. The study revealed significant variation among the genotypes for LLS and rust resistance at different environments. Combined analysis of variance revealed significant environment (E) and genotype × environment (G×E) interactions for both the diseases indicating differential response of genotypes in different environments. The present study reported 31 genotypes as resistant to LLS and 66 to rust across the locations at 90 DAS with maturity duration 103 to 128 days. Twenty-eight genotypes showed resistance to both the diseases across the locations, of which 19 derived from *A. cardenasii*, five from *A. hypogaea*, and four from* A. villosa.* Site regression and Genotype by Genotype x Environment (GGE) biplot analysis identified eight genotypes as stable for LLS, 24 for rust and 14 for pod yield under disease pressure across the environments. Best performing environment specific genotypes were also identified. Nine genotypes resistant to LLS and rust showed 77% to 120% increase in pod yield over control under disease pressure with acceptable pod and kernel features that can be used as potential parents in LLS and rust resistance breeding. Pod yield increase as a consequence of resistance offered to foliar fungal diseases suggests the possibility of considering ‘foliar fungal disease resistance’ as a must-have trait in all the peanut cultivars that will be released for cultivation in rainfed ecologies in Asia and Africa. The phenotypic data of the present study will be used for designing genomic selection prediction models in peanut.

## Introduction

Peanut (*Arachis hypogaea* L.) is an important annual food, feed, and oilseed crop grown nearly in 114 tropical and subtropical countries, covering an area of 27.66 m ha, annual production of 43.98 m tonne and productivity of 1590 kg/ha (FAOSTAT, 2016). The productivity of peanut in Asia (2186 kg/ha) and Africa (903 kg/ha) are quite low in comparison to America (3381 kg/ha), Europe (3102 kg/ha), and Australia and New Zealand (2825 kg/ha) (FAOSTAT, 2016). Exposure to various biotic and abiotic stresses, poor agronomic management practices, non-availability of quality seeds of released varieties and socio-economic issues are some key factors for the low productivity in Asia and Africa. Among the biotic stresses, foliar diseases such as late leaf spot (LLS) (caused by *Phaeoisariopsis personata* Berk. & Curtis) and rust (caused by *Puccinia arachidis* Speg.) are economically most important. Nearly 50–70% reduction in pod yield and adverse effect on seed quality was reported due to infection of rust and LLS together (Miller et al., 1990; Grichar et al., 1998). Plants susceptible to LLS exhibit complete defoliation under high disease pressure leading to low yield. Leaf rust also has considerable economic importance in many peanut growing regions of the world. The losses due to occurrence of rust can vary from 40% to 70% under favorable conditions and presence of susceptible cultivars (Subrahmanyam et al., 1985; Dwivedi et al., 2002). The disease can be particularly severe when it occurs together with LLS.

Identifying disease resistant genotypes and introgressing trait into the improved genetic background is one of the most effective and eco-friendly measures to enhance production and productivity under resource-limited farming systems especially in semi-arid regions of developing countries. In the past, several efforts were made to identify sources of resistance to LLS (Subrahmanyam et al., 1985; Gorbet et al., 1990) and rust (Wynne et al., 1991; Subrahmanyam et al., 1989) in peanut. Majority of identified resistant sources belong to subspecies *fastigiata* var. *fastigiata* and are landraces from South America (Subrahmanyam et al., 1989). Wild *Arachis* species, in contrast, have shown variation ranging from immune to highly resistant reaction to LLS (Abdou et al., 1974; Subrahmanyam et al., 1985). However, the use of wild species in resistance breeding programs remained limited due to cross-compatibility barriers, the occurrence of linkage drag, late maturity, and undesirable pod and seed features.

Foliar fungal disease screening under field conditions is cumbersome, time-consuming, resource intensive, and often demanding to evaluate large number of individuals of segregating generations. The efficiency and accuracy of selection are largely depending on the environment of disease development and evaluation techniques. Genomic selection (GS) is an emerging approach to increase selection intensity, accuracy, and genetic gains in breeding program for improving complex polygenic traits through increasing frequency of favorable alleles in advance generation with the help of genomic estimated breeding value (GEBV) predicted using whole genome marker profile data and multi-environmental phenotypic data (Meuwissen et al., 2001). An earlier study using Marker Assisted Backcrossing (MABC) approach to introgress a major quantitative trait loci (QTL) explaining 80% Phenotypic Variation (PV) for rust resistance and 65% PV for LLS resistance has revealed that phenotyping for disease resistance together with selection for the QTL of interest is needed to derive lines with the desired level of resistance (Janila et al., 2016). Therefore, GS may be a valuable approach for improving resistance to foliar fungal diseases in peanut as it enables simultaneous selection of several genomic regions based on GEBVs. To implement GS, multi-environment phenotypic and genome-wide markers data on a diverse set of genotypes called genomic selection training population (GSTP) are used to train a prediction model which is applied to a new set of selection candidates that have been genotyped with genome-wide markers. GS using only molecular information prior to phenotyping will be useful for increasing the rate of genetic gain by reducing the breeding cycle time and increasing the selection intensity and accuracy. Therefore, the present study was aimed to evaluate GSTP for resistance to LLS and rust diseases across different environments which will be used for construction GS prediction models in peanut. The present study is the first comprehensive field evaluation of GSTP against rust and LLS diseases. The screening of this diverse set of genotypes for both the diseases also identified genotypes resistant to both diseases which can be used in future breeding programs.

## Materials and Methods

### Plant Material

A set of 340 peanut genotypes, of which 227 belonged to subspecies *fastigiata* and 113 to subspecies *hypogaea*, and differing for morphological and economically important traits constituted a genomic selection training population (GSTP) at ICRISAT. Among the 227 genotypes of ssp. *fastigiata*, 212 genotypes belong to botanical variety *vulgaris* (Spanish bunch), 10 to *fastigiata* (Valencia), four to *peruviana* and a single genotype to *aequatoriana*; while among the 113 genotypes of ssp. *hypogaea*, 111 genotypes belong to botanical variety *hypogaea*, one to *hirsuta* and one to unknown botanical type. A total of 51 genotypes were taken from 20 different countries whereas 289 were developed/originated at 11 major peanut breeding centers of India. Among these, 189 genotypes were contributed by ICRISAT and 63 by University of Agricultural Sciences, Dharwad. The details of genotypes such as subspecies, botanical variety, market type, origin, and pedigree are given in **Supplementary Table 1**.

### Experimental Design

The experiment was conducted at three locations in India *viz*., International Crops Research Institute for the Semi-Arid Tropics (ICRISAT), Patancheru, Telangana (17º53 ‘N, 78º27 ‘E, 545.0 MSL), Oilseed Research Station (ORS), Mahatma Phule Krishi Vidyapeeth, Jalgaon, Maharashtra (21°03 ‘N, 75°34 ‘E, 201.2 MSL) and Coconut Research Station (CRS), Tamil Nadu Agricultural University (TNAU), Aliyarnagar, Tamil Nadu (10°29 ‘N, 76°58 ‘E, 288.0 MSL) during rainy season 2015 for multi-location evaluation of GSTP against two major foliar fungal diseases (rust and LLS), and pod yield under disease pressure. Nutritional quality traits were assessed during post-rainy 2015–16 at ICRISAT, Patancheru. Two of the evaluation sites, ORS, Jalgaon and CRS, Aliyarnagar are natural disease hotspots for LLS and rust, respectively. At ICRISAT, Patancheru, the natural infection is supplemented with artificial disease infection created by inoculating the diseases through infector row technique. The trials were planted in Alpha Lattice Design with two replications at all the environments. Each replication was divided into 20 equal sized homogeneous blocks with the block size of 17 plots to reduce heterogeneity in the experiments by eliminating inter-block effect. Single row plots were planted with 4 m length and with inter and intra-row spacing of 30 and 10 cm, respectively. The sowing was done on broad bed system as recommended for peanut cultivation with 4 rows per bed. Standard agronomic management practices were followed at each environment: 60 kg phosphorus pentoxide (P_2_O_5_) as a basal application, pre-emergence application of Pendimethalin (@1 kg active ingredient per ha) for weed control and irrigation soon after planting and subsequently when needed. There were no disease symptoms observed during the post-rainy season, hence management practices were not adopted for either of the diseases. Gypsum (@400 kg/ha) was applied to the experimental field at peak flowering stage and protection was taken against insects whereas no protection measure applied to control foliar fungal diseases.

### Field Evaluation of GSTP for Resistance to LLS and Rust

At Aliyarnagar and Jalgaon which are natural hotspots for rust and LLS, infector rows of a highly susceptible cultivar TMV 2 were planted after every four broad beds to maintain uniform disease pressure. At ICRISAT, artificial disease screening was used with infector rows of TMV 2 after every four broad beds, and along the borders to create optimum disease pressure for screening. For artificial inoculation, urediniospores of *Puccinia arachidis* (rust) and conidial suspension of *Phaeoisariopsis personata* (LLS pathogens) were collected separately using a cyclone spore collector (Fischer Scientific Co., USA) from naturally infected leaf lesions of the susceptible cultivar TMV 2. The inoculum were stored at −20°C. Ten days before field planting, the susceptible peanut cultivar TMV 2 was planted in polybags in the greenhouse. Thirty-five day-old TMV 2 seedlings raised in the greenhouse were inoculated separately by spraying with *urediniospores* of rust and conidia of LLS at 5 × 10^4^ spores ml^−1^. The non-ionic detergent, Tween 20 was added to the spore solution as a surfactant at the rate of 0.05% of the spore solution. Water was sprinkled in and around the inoculated plants in the polybags and the plants were covered with polyethylene sheet during the nights for 7 days to maintain high humidity (95%). Severe rust and LLS developed on these plants in two weeks. The infected plants in polybags were transplanted in the infector rows of the trial at one-meter distance around 50 days after sowing (DAS). Conidia of LLS and *urediniospores* of rust were sprayed at a concentration of 5×10^4^ spores ml^−1^ on infector rows of the trial. Sprinkler irrigation was provided to the trial daily for 30 min for a period of one month starting from the day of field inoculation with the pathogen to promote disease development (Sudini et al., 2015).

### Observations

The visual disease scoring on a modified 1 to 9 point scale for LLS and rust given by Subrahmanyam et al. (1995) was used for recording disease scores at three different crop growth stage *viz*., 75, 90, and 105 DAS or at harvest for the entries maturing in <105 days. This is a standard procedure for recording disease scoring for genotypes of medium maturity group (100 to 130 days). The disease severities corresponding to the rust and LLS scores are 1 = 0%; 2 = 1–5%; 3 = 6–10%; 4 = 11–20%; 5 = 21–30%; 6 = 31–40%; 7 = 41–60%; 8 = 61–80%; and 9 = 81–100%. Based on the disease severity scores at 90 and 105 DAS, genotypes were categorized into resistant (≤3), moderate resistance (4–5), susceptible (6–7), and highly susceptible (>7) (Sudini et al., 2015). Genotypes with lowest severity ratings for LLS and rust at ICRISAT and Aliyarnagar were selected for evaluating the disease progress at 75, 90, and 105 DAS and were compared with that of resistant and susceptible checks. Days to maturity, hundred kernel mass and pod yield per hectare was also recorded across the environments. Haulm yield per plant was only recorded at ICRISAT during rainy 2015.

### Statistical Analysis

Standard statistical procedures were adopted for data analysis. Individual, as well as combined analysis of variance (ANOVA) was computed using general linear mixed model using proc glm function of SAS version 9.2 (SAS Institute Inc, 2008). Best linear unbiased predictions or adjusted means were estimated for every trait except disease severity scores of rust and LLS because higher severity score among both the replications was considered as the final score of genotype. Test for the homogeneity of error variances was conducted for disease severity scores and yield traits using Levene’s test (Steel and Torrie, 1980). Genotypes which had ≤3 disease severity scores for either of the diseases at ICRISAT_R15 were selected to check their stability for reaction against both the diseases and pod yield across the environments. The stability analysis of 110 selected genotypes for disease reaction against LLS and rust at 90 DAS was done using the data recorded in rainy season 2015 across three locations whereas for pod yield, data recorded during post-rainy 2015–16 data was also used in analysis.

Site regression analysis (commonly known as GGE biplot) was used to illustrate the genotype plus genotype-by-environment variation using principal components (PC) scores from singular value decomposition (SVD) (Yan et al., 2000). GGE biplot with average-environment coordination (AEC) and polygon view was drawn to examine the performance of all genotypes within a specific environment and to simultaneous select genotypes based on stability and mean performance. The model for the GGE based on SVD of first two PCs is given by:

$$Y_{ij}-\mu -\beta_{j}=\lambda_{1}\xi_{i1}\eta_{j1}+\lambda_{2}\xi_{i2}\eta_{j2}+\varepsilon_{ij}$$

Where *Y* *_ij_* is the mean performance of genotype i in environment *j*,µ is the grand mean, β*_j_*is the environment *j *main effect, λ_1_ and λ_2_ are the singular values of the first and second PC, ξ*_i_*_1_ and ξ*_i_*_2_ are the eigenvectors for genotype i*,* and η*_j_*_1_ and η*_j_*_2_ are the eigenvectors for environment *j* and ε*_ij_* is the residual effect. Simple scatter plot was also plotted for comparing environment-centered incidence score of genotypes in two environments. All analyses were performed using GenStat software 15th edition (VSN International, Hemel Hempstead, UK).

## Results

### Analysis of Variance and Genetic Variability Parameters for Disease Resistance

Individual environment ANOVA revealed significant genotypic differences (*p* < 0.001) for LLS and rust disease score at 90 DAS, days to maturity and pod yield per hectare under disease pressure (rainy 2015) and pod yield under the absence of disease pressure (Post-rainy 2015–16) (data not presented). Combined ANOVA showed significant genotypic differences along with significant environment and genotype × environment (G×E) interaction (GEI) effects (*p* < 0.001) for LLS and rust disease score at 90 DAS, days to maturity and pod yield per hectare under disease pressure and disease free condition. The environmental variance was high for both diseases. The genotypic variance was high compared to G×E interaction variances (**Table 1**).

**Table 1:** Combined analysis of variance for disease score of LLS and rust across the environments during rainy season 2015.

Source	df^a^	LLS75	LLS90	LLS105	Rust75	Rust90	Rust105	df^b^	DM	PYH
**Environment**	2	12.554**	12.97**	10.174**	10.496**	10.853**	27.029**	3	167711.34**	7700211.4**
**Replication (ENV)**	3	0.208**	0.078**	0.074**	0.125**	0.102**	0.219**	4	634.68**	4321173.2**
**Block (ENV × REP)**	114	0.005	0.008**	0.008**	0.004	0.01**	0.011**	152	2.87	159853.1
**Genotypes**	339	0.024**	0.042**	0.03**	0.04**	0.068**	0.059**	339	274.45**	2105123.5**
**Genotype × Environment**	678	0.009**	0.008**	0.008**	0.014**	0.011**	0.011**	1017	41.93**	590881.7**
**Error**	903	0.004	0.003	0.004	0.004	0.005	0.006	1204	3.15	110685.0

Where ** represents significant at 1% probability level.

df^a^, Degree of freedom for LLS75, LLS90, LLS105, Rust75, Rust90, Rust105; df^b^, Degrees of freedom for days to maturity and pod yield per hectare; LLS75, LLS90, and LLS105, Disease severity score of late leaf spot recorded at 75, 90, and 105 days after sowing, respectively, and Rust75, Rust90, and Rust105, Disease severity score of rust recorded at 75, 90 and 105 days after sowing, respectively; DM, Days to maturity; PYH, Pod yield per hectare; ENV, Environment; REP, Replication.

The estimates of genetic variability parameters revealed high genetic variability for rust and LLS at 75, 90, and 105 DAS (**Table 2**). In general, the phenotypic coefficient of variation (PCV) was higher than the genotypic coefficient of variation (GCV) across individual environments and pooled analysis. The GCV and PCV values were moderate at 90 DAS and low at 105 DAS. High estimates of heritability in broad sense for rust (82.0%) and LLS (80.9%) disease score at 90 DAS coupled with high genetic advance as percent of mean (GAM) (28.2% for rust and 21.2% for LLS) was reported across the environments.

**Table 2:** Mean, range, and genetic parameters for disease severity scores to LLS and rust on GSTP of peanut evaluated across the locations during rainy season 2015.

Traits	Mean	Range		GCV (%)	PCV (%)	h^2^_bs _(%)	GAM (%)
		Min	Max				
**Aliyarnagar rainy 2015**							
LLS 75	1.9	1	4	17.06	23.65	52.00	25.34
LLS 90	4.6	2	8	15.14	16.53	83.86	28.55
LLS 105	7.1	3	9	7.54	10.40	52.64	11.27
Rust 75	2.4	1	5	26.43	30.66	74.29	46.93
Rust 90	4.9	1	8	19.67	21.22	85.95	37.57
Rust 105	6.9	2	9	9.96	12.05	68.26	16.95
**Jalgaon rainy 2015**							
LLS 75	1.1	1	3	2.24	10.78	4.34	0.96
LLS 90	3.3	1	6	12.84	17.44	54.17	19.46
LLS 105	4.8	2	8	13.35	16.40	66.30	22.39
Rust 75	1.0	1	3	4.01	5.35	56.08	6.18
Rust 90	3.0	1	6	14.20	20.39	48.54	20.39
Rust 105	4.3	2	8	23.73	31.01	58.57	37.41
**ICRISAT rainy 2015**							
LLS 75	3.3	1	6	17.12	20.74	68.09	29.09
LLS 90	6.7	2	9	10.13	11.28	80.76	18.76
LLS 105	8.2	4	9	7.71	8.53	81.55	14.34
Rust 75	3.1	1	6	21.36	25.09	72.43	37.44
Rust 90	5.8	2	8	14.73	16.15	83.27	27.70
Rust 105	7.7	3	9	10.41	11.43	82.89	19.53
**Pooled across the locations **							
LLS75	2.1	1	4	13.62	16.68	66.61	22.89
LLS90	4.9	2	7	11.42	12.70	80.90	21.17
LLS105	6.7	4	8	8.06	9.42	73.25	14.21
Rust75	2.1	1	4	17.19	21.51	63.82	28.28
Rust90	4.6	2	7	15.13	16.71	82.00	28.22
Rust105	6.3	3	8	12.52	13.96	80.45	23.13

LLS75, LLS90, and LLS105 = Disease severity score of late leaf spot at 75, 90 and 105 days, respectively; Rust75, Rust90, and Rust105 = Disease severity score of rust at 75, 90 and 105 days, respectively; Min, Minimum; Max, Maximum; GCV, Genotypic co-efficient of variation (%); PCV, Phenotypic co-efficient of variation (%); h^2^_bs_, Heritability in broad sense (%); GAM, Genetic advance as percent of mean (%).

### Disease Reaction of Genotypes Against LLS

The disease pressure was high for LLS and rust at Aliyarnagar and ICRISAT as observed by the disease severity score of ≥8 for the susceptible cultivar TMV 2 at 90 DAS. Moderate disease pressure was observed at Jalgaon wherein a disease severity score of 5 was recorded on TMV 2 at 90 DAS for LLS and rust.

**Figure 1:**
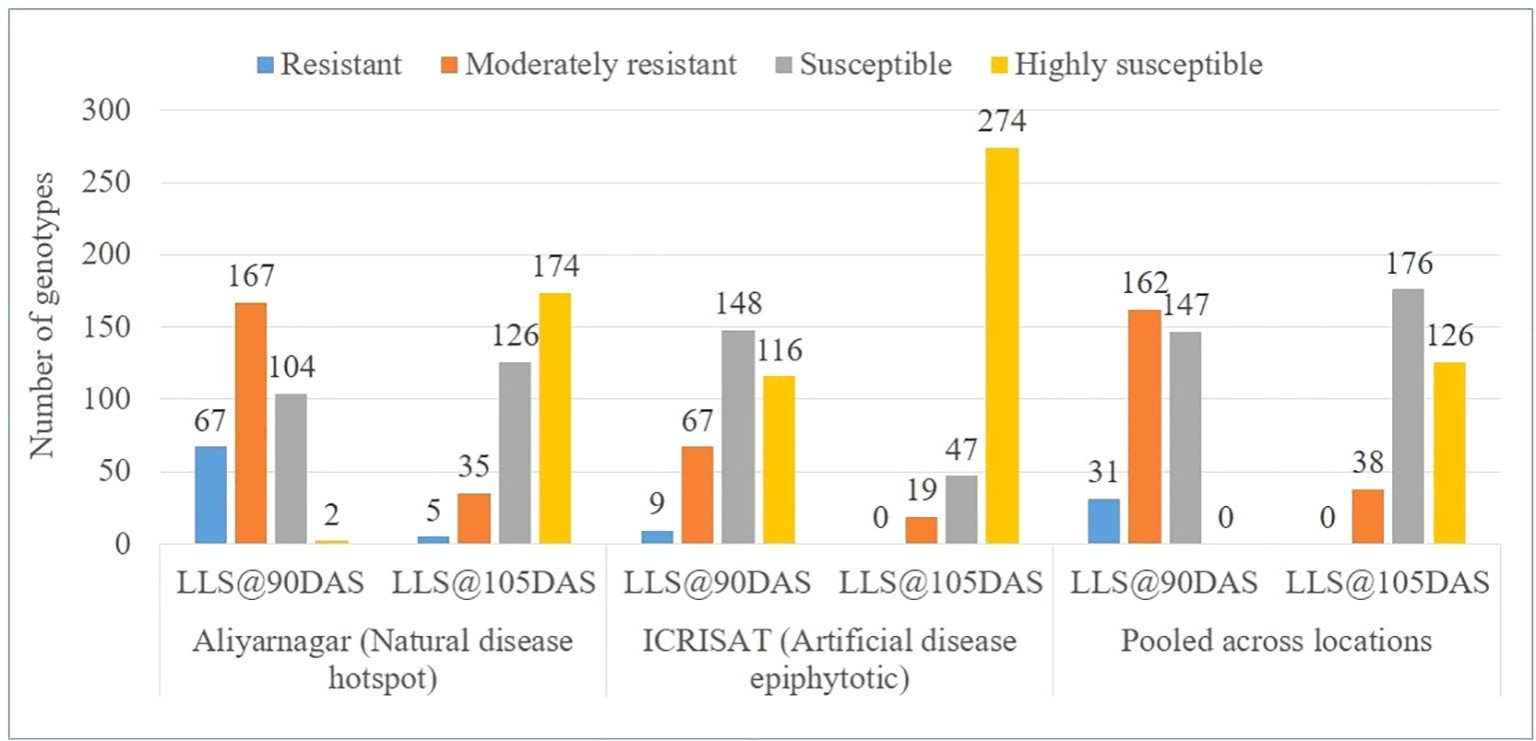
Categorization of genotypes based on reaction against LLS at 90 and 105 days after sowing (DAS) at Aliyarnagar, ICRISAT and pooled across the environments during rainy season 2015.

The disease score of genotypes for LLS at ICRISAT varied from 1 to 6 at 75 DAS, 2 to 9 at 90 DAS and from 4 to 9 at 105 DAS. However, at Aliyarnagar it varied from 1 to 4 at 75 DAS, 2 to 8 at 90 DAS and from 3 to 9 at 105 DAS; whereas at Jalgaon it varied from 1 to 3 at 75 DAS, 1 to 6 at 90 DAS, and from 2 to 8 at 105 DAS (**Table 2**). Due to moderate disease pressure at Jalgaon, the genotypes were not categorized into resistant and susceptible groups. Out of 340 genotypes of GSTP, 67 reported as resistant (R), 167 as moderately resistant (MR), 104 as susceptible (S) and two genotypes as highly susceptible (HS) to LLS at 90 DAS whereas, five genotypes exhibited R, 35 MR, 126 S, and 174 HS reaction to LLS at 105 DAS at Aliyarnagar (**Figure 1**). Out of five resistant lines, four were matured in >115 days whereas one line SPS 7 matured in 104 days. At ICRISAT, nine R, 67 MR, 148 S, and 116 HS genotypes to LLS at 90 DAS at ICRISAT (**Figure 1**). Of the nine resistant lines, only one (ICGV 86699) matured in <100 days whereas eight other lines matured in >120 days with disease score of 4 to 5 at 105 DAS. None of the genotypes showed resistant reaction to LLS up to 105 DAS at ICRISAT, while 19 genotypes had MR, 47 S, and 274 HS reaction to LLS at 105 DAS (**Figure 1**).

The pooled LLS scores varied from 1 to 4 at 75 DAS, 2 to 7 at 90 DAS, and 4 to 8 at 105 DAS. Thirty-one genotypes showed R, 162 MR, and 147 S reaction against LLS at 90 DAS across the environment (**Figure 1** and **Supplementary Table 3**). None of the genotypes of GSTP showed R reaction against LLS up to 105 DAS while 38 identified as MR, 176 as S, and 126 as HS to LLS at 105 DAS across the environment (**Figure 1**). Out of moderately resistance genotypes, 7 matured in ≤120 days where 31 other matured in >120 days.

At ICRISAT, 283 out of 340 genotypes matured in <120 days whereas remaining 57 genotypes matured in >120 days. Of the 283 genotypes, ICGV 86699 showed resistance to LLS at 90 DAS with disease score of 2, whereas four other lines, ICGVs 01273 and 00362, SPS 2, and SPS 8 were moderately resistance with disease score of 4 for LLS at 90 DAS. Nineteen genotypes showed resistance to rust with a score of ≤3 at 90 DAS. Out of 57 genotypes that matured later (>120 days), eight recorded a disease score of ≤3 at 90 DAS, and four to five at 105 DAS. Sixteen genotypes were moderately resistant to LLS with disease score of 4 to 5 at 105 DAS. However, nine genotypes showed a resistant reaction to rust with ≤3 disease score at 90 DAS and 3 to 5 at 105 DAS.

### Disease Reaction of Genotypes Against Leaf Rust

**Figure 2:**
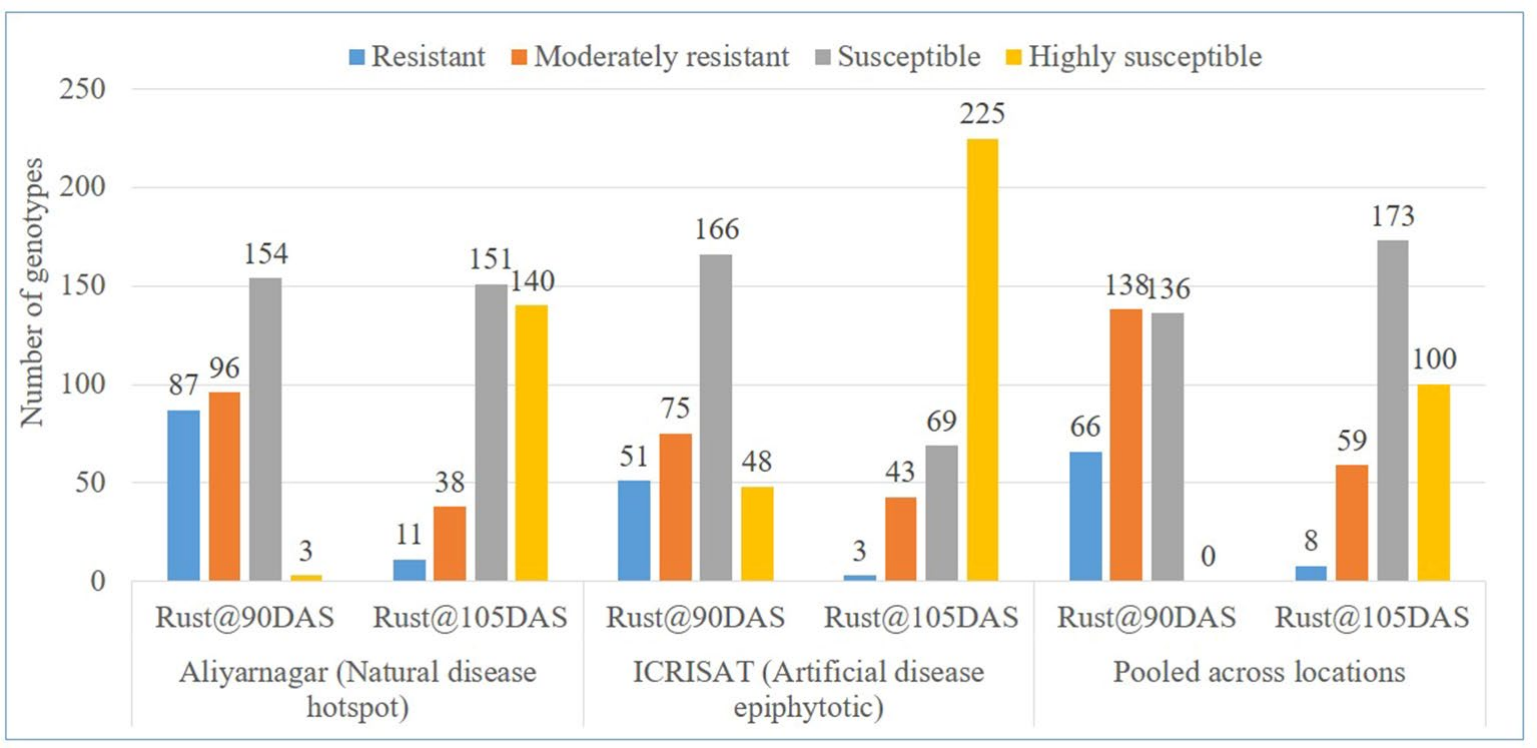
Categorization of genotypes based on reaction against rust at 90 and 105 DAS at Aliyarnagar, ICRISAT and pooled across the environments during rainy 2015.

The disease severity scores of genotypes for rust at Aliyarnagar varied from 1 to 5 at 75 DAS, 1 to 8 at 90 DAS, and 2 to 9 at 105 DAS. At Jalgaon, the rust score varied from 1 to 3 at 75 DAS, 1 to 6 at 90 DAS, and 2 to 8 at 105 DAS. However, disease severity scores of genotypes for rust under artificial disease pressure at ICRISAT varied from 1 to 6 at 75 DAS, 2 to 8 at 90 DAS, and 3 to 9 at 105 DAS (**Table 2**). Out of 340 genotypes of GSTP, 87 exhibited R, 96 MR, 154 S and 3 HS reaction against rust at 90 DAS whereas 11 genotypes were reported with R, 38 with MR, 151 with S, and 140 with HS reaction against rust at 105 DAS at Aliyarnagar (**Figure 2**). However, 51 genotypes reported as R, 75 as MR, 166 as S and 48 as HS to rust at 90 DAS under artificial disease pressure at ICRISAT (**Figure 2**). Three genotypes showed resistant reaction against rust up to 105 DAS while 43 genotypes were reported as MR, 69 as S, and 225 as HS to rust at 105 DAS under artificial disease pressure at ICRISAT during rainy 2015 (**Figure 2**).

The disease severity scores of genotypes for rust across the environments varied from 1 to 4 at 75 DAS, 2 to 7 at 90 DAS, and 3 to 8 at 105 DAS. Out of 340 genotypes, 66 exhibited R, 138 MR, and 136 S against rust at 90 DAS across the environments (**Figure 2** and **Supplementary Table 4**). However, eight genotypes showed R, 59 MR, 173 S, and 100 HS reaction against rust across the environments at 105 DAS (**Figure 2**).

### Stability of Disease Reaction Across the Environments

**Figure 3:**
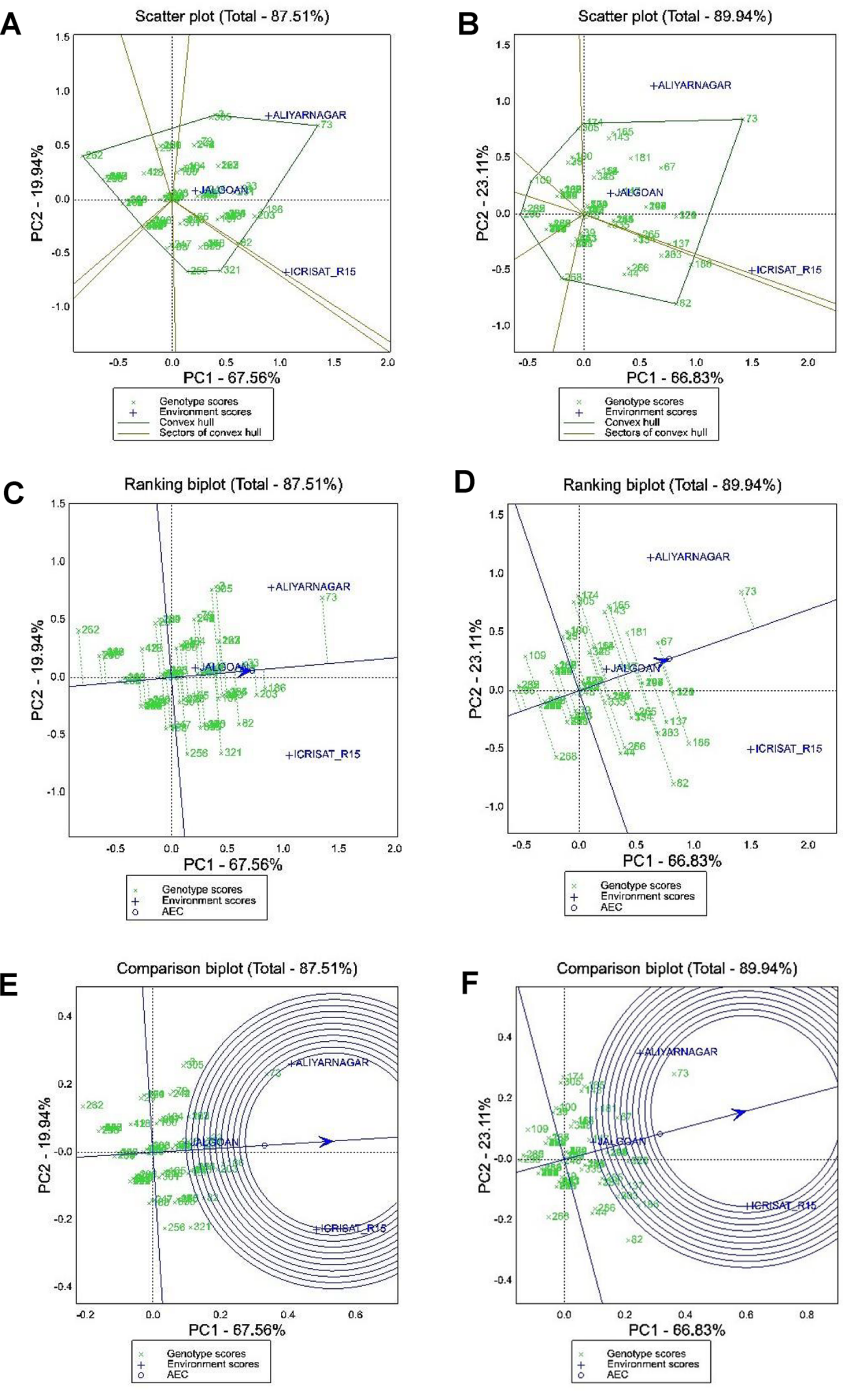
GGE biplot analysis for disease severity scores to LLS and rust at 90 DAS. **(A** and **B)** polygon view of scattered biplot showing ranking of genotypes based on which won where pattern for disease severity against late leaf spot and rust, respectively; **(C** and **D)** GGE biplot showing ranking of genotypes for mean and stability of disease severity scores of LLS and rust, respectively; **(E** and **F)** Comparison of environments with respect to ideal test environment for disease severity of LLS and rust, respectively. Area of inner circle of in biplot represents ideal test environment and the environment plotted within this circle are the best environments for cultivar evaluation.

Out of 340 genotypes of GSTP evaluated for resistance to rust and LLS along with yield traits, 109 genotypes which had ≤3 disease severity score for rust and LLS at ICRISAT and Aliyarnagar along with a susceptible check (TMV 2) were subjected to stability analysis to identify stable sources of disease resistance and pod yield. The GGE biplot graphically explains genotype main effect along with genotype × environment interaction using first two principal components (PC1 and PC2) derived from SVD of the environment-centered data. The first two PCs in the biplot (PC1 and PC2) explained 87.51% and 89.94% of the total variation due to genotype main effect and GEI for LLS and rust at 90 DAS, respectively (**Figure 3**).

#### (a) Polygon View of GGE Biplot for LLS and Rust Scores at 90 DAS

The polygon view of a biplot is the best way to visualize the interaction patterns between genotypes and environments to show the presence or absence of crossover GEI which is helpful in estimating the possible existence of different mega-environments. Visualization of the “which won where” pattern of MET data is necessary for studying the possible existence of different mega-environments in the target environment. In the biplot presented in **Figures 3A, B**, a polygon was formed by connecting the vertex genotypes with straight lines and the rest of the genotypes placed within the polygon. For LLS score at 90 DAS the vertex genotypes were 262, 238, 3, 73, 186, 269, 82, 321, 256, and 268 (**Figure 3A**). These genotypes were the best or the poorest genotypes for disease resistance in some or all of the environments because they were farthest from the origin of the biplot. From the polygon view of biplot analysis of MET data in three environments, the genotypes fell in four sections and the test environments fell in two sections. The first section contains the test environments Aliyarnagar and Jalgaon and the vertex genotypes for this section were genotype 73 (TMV 2) which is susceptible to LLS whereas genotype 262 (ICGV 86699) plotted farthest on the left side indicates lowest disease scores across the environments. The second section contains the environments ICRISAT_R15 (ICRISAT rainy season 2015) with the genotype 321 (ICG 13895) as the high scoring genotype for LLS.

Similarly, for rust score at 90 DAS the vertex genotypes were 296, 109, 305, 174, 73, 186, 82, and 268 (**Figure 3B**). These genotypes were the best or the poorest genotypes for rust resistance in some or all of the environments because they were farthest from the biplot origin on either of the sides. For rust, the genotype 73 (TMV 2) plotted farthest on the right side of the biplot indicating its high susceptibility, whereas genotypes 236 (ICGV 99052) and 301 (ICG 11426) which plotted farthest on the left side of biplot were resistant across the environments.

#### (b) Mean Performance and Stability of Genotypes for LLS and Rust Score at 90 DAS

The ranking of 109 genotypes of GSTP based on their disease severity score and stability performance for LLS and rust have been presented in **Figures 3C, D**, respectively. The line passing through the biplot origin is called the average environment axis (AEA), which is defined by the average PC1 and PC2 scores of all environments. A concentric circle drawn on AEA is called AEC. The genotypes closer to concentric circle indicates higher mean performance. The line which passes through the biplot origin and is perpendicular to the AEA represents the stability of genotypes. Distance in either direction away from the biplot origin on this axis indicates greater GEI and reduced stability. The genotypes on the right side of this perpendicular line performed greater than mean disease severity score across the environments and the genotypes on the left side of this line had lesser score than mean across the environments. In the biplot, the genotypes plotted left side of biplot and have the shortest vector from the AEA are better genotypes. For selection, the stable resistant genotypes are those with both lowest disease severity score and least vector length from AEA. The genotype 71 (GPBD 4), 238 (ICGV 00248), 84 (ICGV 06142), 152 (ICGV 02411), 237 (ICGV 00246), 246 (ICGV 00068), 293 (SPS 11), and 301 (ICG 11426) were found as stable resistant genotypes based on their disease score for LLS and vector length from AEA (**Figure 3C**). The genotype 262 (ICGV 86699) had lowest disease score of LLS compared to others with greater vector length from AEA.

For rust the genotypes 236 (ICGV 99052), 301 (ICG 11426), 235 (ICGV 99051), 262 (ICGV 86699), 71 (GPBD 4), 27 (ICGV 06422), 30 (ICGV 07223), 32 (ICGV 07235), 77 (ICGV 05100), 84 (ICGV 06142), 152 (ICGV 02411), 153 (ICGV 05155), 229 (ICGV 00362), 237 (ICGV 00362), 238 (ICGV 00248), 239 (ICGV 01361), 252 (ICGV 99160), 253 (ICGV 02323), 260 (ICGV 87846), 288 (SPS 2), 291 (SPS 7), 293 (SPS 11), 296 (SPS 21), and 303 (ICGV 02446) can be considered as stable resistant genotypes based on their low disease score and short vector length (**Figure 3D**). Also, the genotype 109 (49 M-16) and 268 (ICGV 05032) had lower mean disease score for rust but greater vector length from AEA indicating their unstable nature. Genotype 109 recorded high disease score at Aliyarnagar whereas 268 recorded high disease score at ICRISAT.

#### (c) Relationship Among Test Environments

The summary of the interrelationships among the test environments for LLS and rust has been presented in **Figures 3E, F**, respectively. The lines that connect the biplot origin and the markers for the environments are called environment vectors. The angle between the vectors of two environments is related to the correlation coefficient between them. The cosine of the angle between the vectors of two environments approximates the correlation coefficient between them. Acute angles indicate a positive correlation, obtuse angles a negative correlation and right angles indicate no correlation. A short vector may indicate that the test environment is not related to other environments. Based on the angles between environment vectors, all the three environments (Aliyarnagar, Jalgaon, and ICRISAT_R15) were positively correlated with each other for LLS and rust because of acute angles (< 90°) formed between them. View of position of environments on biplot revealed that ICRISAT_R15 was the most suitable environment for screening genotypes for LLS and rust followed by Aliyarnagar whereas Jalgaon was the poor environment plotted nearer to biplot origin indicates that genotypes recorded lower disease scores at this environment. Also, the ranking of environments with respect to ideal test environments (**Figures 3E, F**) revealed that the ICRISAT_R15 and Aliyarnagar plotted on the border of inner circle in the biplot indicating that both had similar disease pressure and are ideal for cultivar evaluation against LLS and rust disease.

##### Stability for Pod Yield

**Figure 4:**
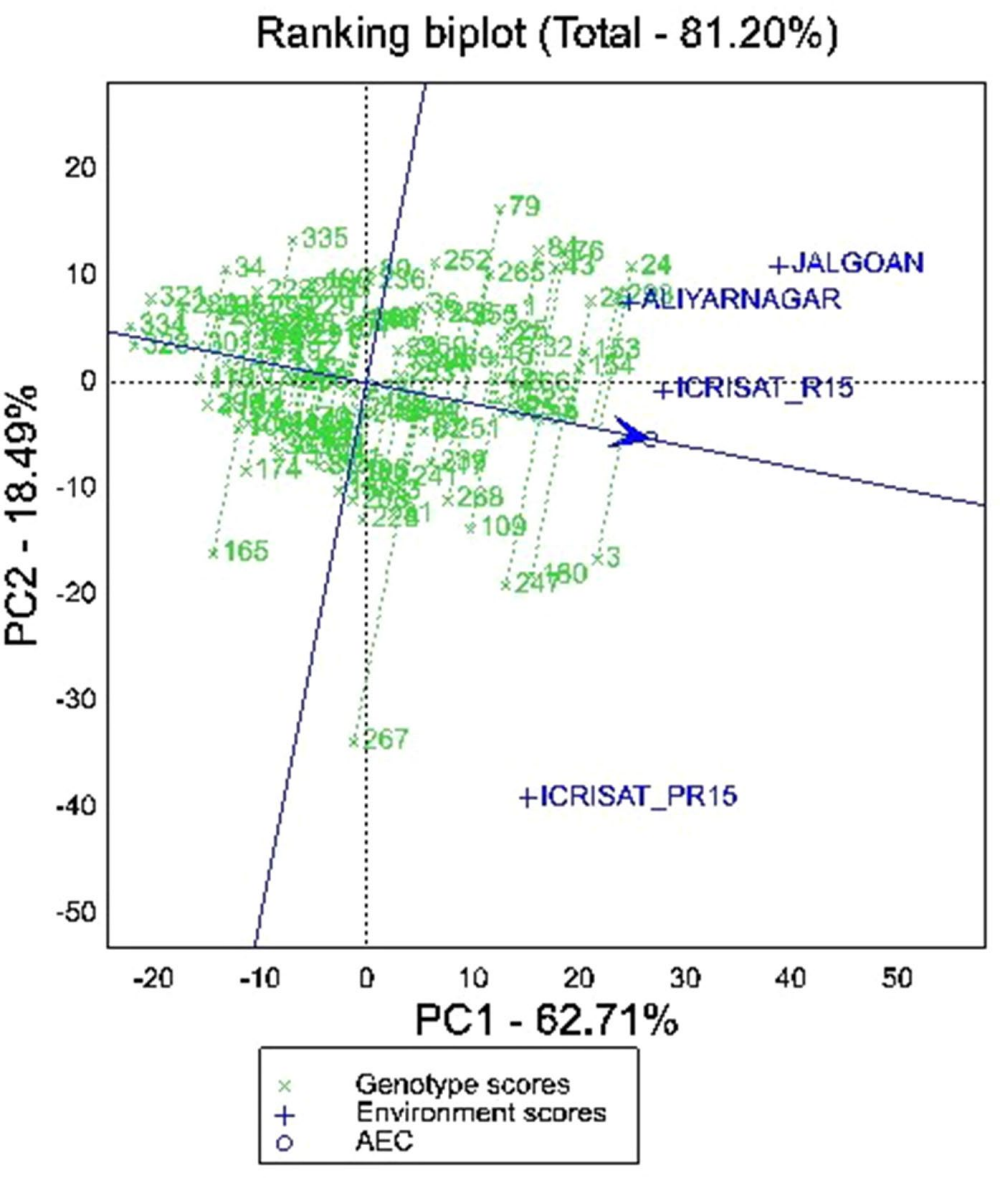
GGE biplot showing ranking of genotypes for mean and stability for pod yield per hectare across the environments.

The partitioning of GEI through GGE biplot analysis showed that PC1 and PC2 together accounted for 81.20% of GGE mean sums of squares for pod yield per hectare (**Figure 4**). The vertex genotypes in the biplot are 79, 24, 293, 3, 267, 165, 328, 334, 321, 34, and 335 indicating that these genotypes were the best or the poorest genotypes for pod yield per hectare in some or all the environments depending on their direction from the origin (**Figure 4**). The polygon view of MET data of four environments in the biplot showed that genotypes fell in four sections whereas the test environments fell into two sections. The first section contains the test environments Aliyarnagar and Jalgaon, whereas the second section contains ICRISAT_R15 and ICRISAT_PR15 (ICRISAT post-rainy season 2015–16). Among these four environments, ICRISAT_PR15 was farthest from the biplot origin followed by Jalgaon, Aliyarnagar and ICRISAT_R15. The distance indicated that the genotypes performed better at ICRISAT_PR 15 followed by Jalgaon.

The ranking biplot of genotypes based on mean pod yield and stability revealed that genotype 154 (ICGV 06100), 26 (ICGV 05163), 153 (ICGV 05155), 30 (ICGV 07223), 32 (ICGV 07235), 253 (ICGV 02323), 266 (ICGV 06099), 37 (ICGV 07120), 152 (ICGV 02411), 25 (ICGV 05161), 45 (ICGV 03043), 1 (ICGV 06423), 42 (ICGV 01273), and 27 (ICGV 06422) were superior and stable performers across the environments. The genotype 3 (ICGV 07247), followed by 24 (ICGV 03064), 293 (SPS 11), 180 (ICGV 01276), 247 (ICGV 01495), 84 (ICGV 06142), 43 (ICGV 01274), 76 (ICGV 03042), 109 (49 M-16), and 268 (ICGV 05032) were also higher yielding genotypes but greater vector length from AEA indicates their unstable performance for pod yield per hectare (**Figure 4**). Among these, 3 (ICGV 07247), 180 (ICGV 01276), 247 (ICGV 01495), and 109 (49 M-16) are plotted near to Aliyarnagar and Jalgaon indicating their environment specific adaptability under these environments whereas 24 (ICGV 03064), 293 (SPS 11), 84 (ICGV 06142), 43 (ICGV 01274), and 76 (ICGV 03042) plotted towards ICRISAT_PR15 indicating their superior performance at ICRISAT during post-rainy season compared to other genotypes (**Figure 4**).

### Discussion

In the present study, significant differences for genotypes, environment and G × E interaction effects was observed for disease scores of LLS and rust at all three stages of growth (75, 90, and 105 DAS) indicating their polygenic nature and the role of genotype, environment and their interaction in disease infection, establishment, and spread. The diverse nature of location and differences in the environmental condition is reflected by mean squares due to environment in ANOVA indicates that the environment plays an important role in these disease traits. The mean squares due to error term represent the unexplained variation in the experiment. The negligible error mean square values for disease traits could be attributed to the precision in conducting experiment and analysis, the robustness of experimental design in explaining/partitioning the total variation into different sources of variation and the unit of measurement. While stable resistance across the growing environment can be identified from the present study, the significant G, E, and G × E interactions for resistance to LLS and rust suggests the possibility of identifying resistance with specific adaptability to a target environment and the need to deploy specifically adapted varieties in future for a more effective genetic control of these diseases. The significant environment and G × E interaction effects on rust and LLS resistance (Iwo and Olorunju, 2009; Mothilal et al., 2010a) and pod yield in peanut are also evident from earlier studies (Makinde and Ariyo, 2011; Upadhyaya et al., 2014). The complex nature of inheritance including the role of polygenes with additive effect for LLS (Nevill, 1982; Jogloy et al., 1987; Wambi et al., 2014) and rust (Singh et al., 1984), and the involvement of maternal genes in the inheritance of LLS was also reported (Janila et al., 2013; Narasimhulu et al., 2013). The comparison of mean pod yield of susceptible (SG) and resistant genotypes (RG) at Aliyarnagar (996 kg in SG and 1981 kg in RG), ICRISAT_R15 (1312 kg in SG and 2329 kg RG), and Jalgaon (1579 kg in SG and 1606 kg in RG) showed yield penalty due to disease incidence of LLS and rust. Both the diseases cause serious damage to the crop with pod yield losses up to 70% in commonly grown susceptible cultivars (Harrison, 1973; Subrahmanyam and McDonald, 1987).

Out of 340 genotypes, a total of 31 (9.1%) genotypes were resistant to LLS across the environments. Of these 15 were from ssp. *fastigiata* var *vulgaris* (Spanish bunch), two from ssp. *fastigiata* var *fastigiata* (Valencia) and 14 from ssp. *hypogaea* var *hypogaea* (Virginia bunch). However, 66 genotypes exhibited resistant reaction against rust across the environments, of which 39 were from ssp. *fastigiata* var *vulgaris*, 26 from ssp. *hypogaea* var *hypogaea* and a single genotype from ssp. *fastigiata* var *peruviana*. A total of 28 genotypes showed resistant reaction against both the diseases with ≤3 disease severity score across the environments at 90 DAS. Among these, 15 genotypes were Spanish bunch type whereas 13 were Virginia bunch type. Nine out of 28 genotypes *viz*., SPS 11, ICGV’s 05163, 01274, 06142, 07235, 02323, 02411, 03043, and 49 M-16 recorded >2500 kg equivalent to 77% to 120% increase in pod yield per hectare over the control (**Table 3**).

**Table 3:** Superior performing genotypes for LLS and rust resistance and other yield traits across the environments during rainy season 2015.

S. No.	Pedigree	Source of resistance	LLS90	Rust90	HKM (g)	DM (days)	PYH (kg/ha)	HYPP (g)
1	ICGV 00068	*A. cardenasii*	2.7	2.7	28.6	126	1714	19.3
2	ICGV 00246	*A. cardenasii*	2.7	2.3	34.4	127	1316	21.8
3	ICGV 00248	*A. cardenasii*	2.3	2.3	31.0	127	1819	20.7
4	ICGV 00362	*A. hypogaea*	3.0	2.3	26.7	111	1470	23.7
5	ICGV 01274	*A. cardenasii*	3.0	2.7	32.7	108	2678	14.5
6	ICGV 02323	*A. cardenasii*	3.0	2.3	39.5	128	2621	17.6
7	ICGV 02411	*A. cardenasii*	2.7	2.3	37.1	125	2562	23.0
8	ICGV 02446	*A. cardenasii*	3.0	2.3	32.9	125	1406	24.0
9	ICGV 03043	*A. hypogaea*	3.0	2.7	36.4	126	2547	15.8
10	ICGV 04087	*A. cardenasii*	3.0	2.7	29.9	126	2146	27.5
11	ICGV 05036	*A. cardenasii*	2.7	2.7	36.6	126	2277	27.6
12	ICGV 05100	*A. cardenasii*	3.0	2.3	33.0	126	1784	20.0
13	ICGV 05141	*A. cardenasii*	2.7	2.7	38.7	125	2163	24.9
14	ICGV 05163	*A. cardenasii*	3.0	3.0	35.5	112	2978	18.2
15	ICGV 06142	*A. cardenasii*	2.7	2.3	29.6	128	2677	18.5
16	ICGV 07235	*A. cardenasii*	3.0	2.3	35.5	119	2668	15.3
17	ICGV 86699	*A. cardenasii*	2.0	2.3	27.0	103	1010	20.4
18	ICGV 99051	*A. cardenasii*	2.7	2.3	34.3	126	1839	25.7
19	ICGV 99052	*A. cardenasii*	3.0	2.0	31.5	126	1822	25.7
20	ICGV 99160	*A. hypogaea*	3.0	2.3	35.2	126	2075	24.0
21	SPS 11	* A. villosa*	2.7	2.3	30.7	127	3130	16.2
22	SPS 2	* A. villosa*	2.7	2.3	31.6	112	1394	27.4
23	SPS 20	* A. villosa*	2.3	2.0	28.0	127	923	24.2
24	SPS 8	* A. villosa*	2.7	2.7	28.7	110	1433	25.3
25	49 M- 1-1	*A. hypogaea*	3.0	2.7	45.8	125	1150	19.4
26	49 M-16	*A. hypogaea*	3.0	2.3	31.5	126	2526	19.4
27	ICG 11337	*A. cardenasii*	2.7	2.7	30.9	126	816	23.5
28	GPBD 4 (RC)	*A. cardenasii*	2.3	2.3	27.3	127	1647	22.0
29	TMV 2 (SC)	*–*	7.0	6.7	30.1	106	1421	12.1

Where LLS90 and Rust90, Disease severity score of late leaf spot and rust across the environments at 90 days after sowing, respectively; HKM, Hundred kernel mass (g); DM, Days to maturity; PYH, Pod yield per hectare (kg); HYPP, Haulm yield per plant (g); SC, Susceptible check; RC, Resistant check.

**Figure 5:**
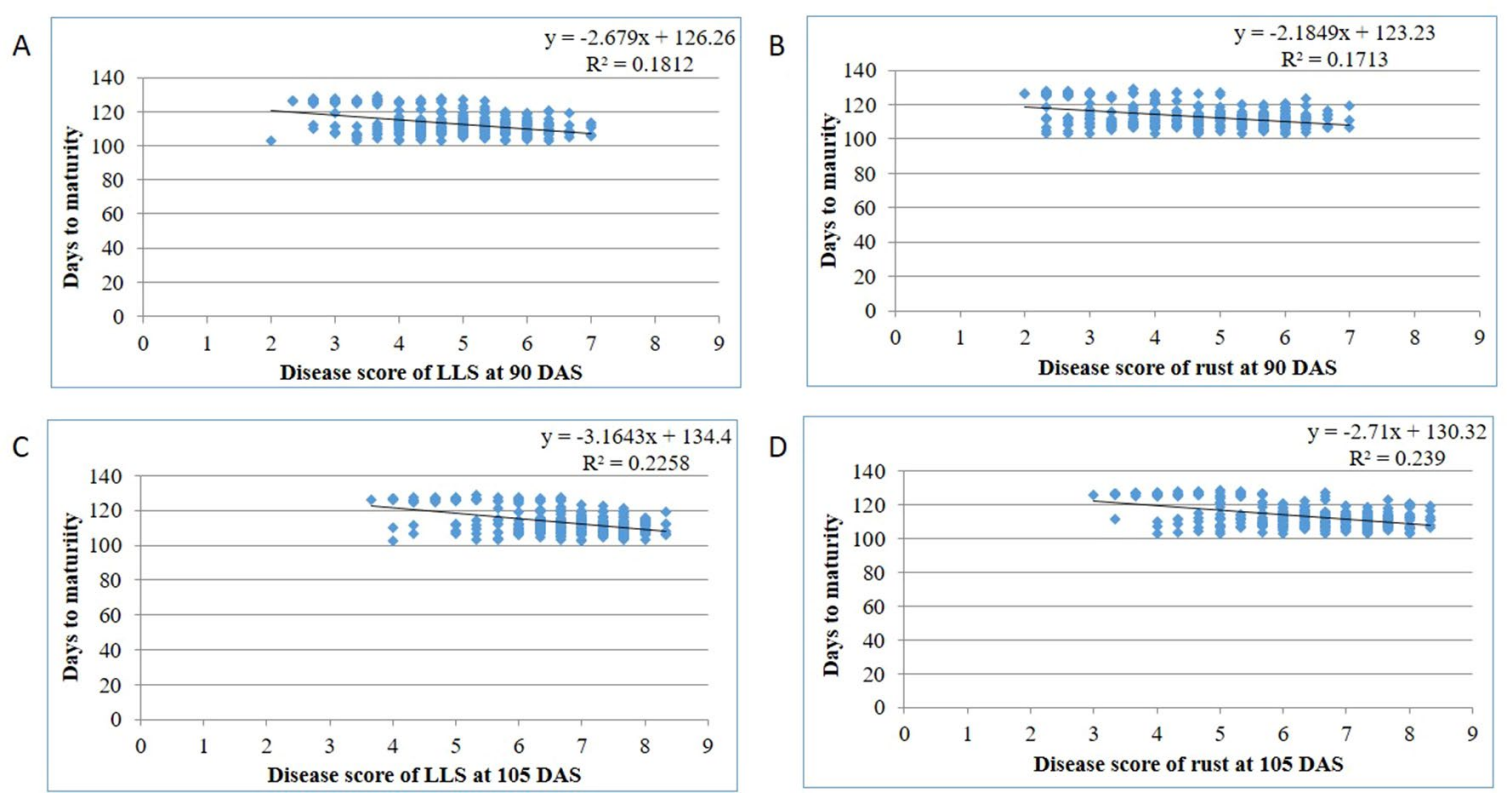
Disease reactions of genotypes for **(A)** LLS at 90 days after sowing (DAS); **(B)** rust at 90 DAS; **(C)** LLS at 105 DAS; **(D)** rust at 105 DAS with respect to days to maturity across the environments during rainy 2015.

Out of 69 resistant genotypes for either rust and LLS or both, 45 belong to *A. cardenasii*, 18 to *A. hypogaea* and 6 to *A. villosa*. A total of 14 genotypes matured in <120 days, of which *A. villosa* derived genotypes had high level of disease resistance to both the diseases (3.00 and 2.50 for LLS and rust at 90 DAS, respectively) followed by *A. cardenasii* (3.67 and 2.77 for LLS and rust at 90 DAS, respectively) and *A. hypogeae* (3.53 and 3.13 for LLS and rust at 90 DAS, respectively). Similarly, out of 55 genotypes that matured in >120 days, *A. villosa* derived genotypes had high level of disease resistance (4.00 and 3.92 for LLS and rust at 105 DAS, respectively) followed by *A. cardenasii* (5.11 and 4.34 for LLS and rust at 105 DAS, respectively) and *A. hypogaea* (5.62 and 4.62 for LLS and rust at 105 DAS, respectively) (**Supplementary Table 2**). In 44 out of 66 genotypes, resistant to rust was derived from *A cardinasii*, in 16 from *A hypogaea* and in six from *A villosa*. Whereas among the 31 genotypes resistant to LLS, 20 had *A. cardenasii* as source of resistance, seven from *A hypogaea* and four from *A. villosa* (SPS 2, SPS 8, SPS 11 and SPS 20). A mutant line M 28-2 belongs to species *hypogaea* used as a source of resistance to develop 49 M-16 and 49 M-1-1 (**Supplementary Table 2**). At Dharwad center, GPBD 4 a popular resistant cultivar was derived from a cross between KRG 1 and ICGV 86855. KRG 1 is an early maturing, Spanish bunch local cultivar susceptible to foliar diseases developed at the Regional Research Station, Raichur, Karnataka through selection from material introduced from Argentine. Whereas, ICGV 86855 (*A hypogaea* x *A. cardenasii*) is a Virginia bunch interspecific derivative, resistant to rust and late leaf spot developed at ICRISAT, Patancheru, India (Gowda et al., 2002a). GPBD 4 and ICGV 86699 derivatives of *A. cardenasii* are the most commonly used sources for rust and LLS resistance breeding programs in India. The identification of resistant lines from the derivatives of *A. villosa* and mutagenesis opens the possibility of widening the genetic base of resistance to both diseases in peanut, which has largely relied on *A. cardinasii* so far. Among the 28 genotypes showed resistance to both the diseases, 20 are advanced breeding lines developed at ICRISAT, seven from University of Agricultural Sciences, Dharwad and a single line from mini-core collection indicating accumulation of favorable alleles for resistant to rust and LLS in the breeding populations. Therefore, recycling the resistant advanced breeding lines results in enhanced genetic gain for resistance to rust and LLS. Resistance in peanut is often associated with long maturity duration (Nigam, 2000). In the present study, the regression analysis showed negative association among disease resistance and maturity duration with a lesser value of the coefficient of determination for LLS (0.18 and 0.24) and rust (0.17 and 0.24) at 90 and 105 DAS, respectively (**Figures 5A–D**). The lines that mature in <120 days, disease score at 90 DAS can be used for comparison among the lines whereas the disease score at 105 DAS should also be considered for the lines that mature in >120 days. Out of five resistance lines identified in Aliyarnagar, four were matured in >115 days whereas one line (SPS 7) matured in 104 days. Similarly, out of nine resistance lines to LLS at 90 DAS, only one (ICGV 86699) has matured in <100 days whereas eight other lines matured in >120 days with disease score of 4–5 at 105 DAS indicates that these lines could resist LLS till 105 DAS. The identified rust and LLS resistant genotypes belonged to early and medium maturity groups (varied from 100 to 130 days) with desirable pod and seed features (**Figures 5A–D**; **Table 3**). Hence, they can be directly utilized in resistance breeding in peanut. Combining foliar fungal disease resistance with early maturity has been a challenge in peanut breeding programs, thus early/medium maturing sources of resistance are preferred by breeders to combine disease resistance with early maturity and high pod yield potential. A few genotypes with early maturity and tolerance to LLS were earlier reported (Branch and Culbreath, 1995). In the present study, GPBD 4, ICGV’s 06142, 02411, 00246, 00248, 00068, 86699, SPS 11, and SPS 20 showed multiple disease resistance with lowest scores for rust and LLS across the environments belong to early and medium maturity group. Genotypes with multiple disease resistance were earlier reported by Gowda et al. (2002a; 2002b), Narasimhulu et al. (2013), and Sudini et al. (2015). The significant G × E interaction effects also create the need to identify stable source of resistance that can perform better under a wide range of environments and/or identify resistance with specific adaptation to a target environment. Being polygenic in nature with background effect, transfer of resistant genes into different backgrounds is quite difficult through conventional breeding (Janila et al., 2013). Hence, it is suggested to use modern tools like genomic selection to overcome the above limitations and realize a higher rate of genetic gain in the breeding programs.

An important objective of resistance breeding is to identify genotypes with durable resistance irrespective of the environment. Horizontal resistance or quantitative resistance is governed by many small effect QTLs or genes with additive effect on resistance mechanism and thus offers more durable resistance compared to vertical major gene resistance. Such type of durable resistance was reported for rust resistance in wheat (Johnsons, 1978), leaf rust of barley (Parlevliet, 1975), stem and leaf rust resistance of wheat (Singh and Rajaram, 1992). Similarly, genetics of LLS and rust resistance in peanut also indicated quantitative inheritance with additive effect of minor genes on inheritance (Janila et al., 2013). Hence, for durable resistance selection for minor gene along with major one should be focused by the breeders. Molecular-assisted selection can assist in the selection of major genes. Major QTLs linked to LLS and rust governing 67% and 80% phenotypic variation were identified in peanut (Khedikar et al., 2010; Sujay et al., 2012; Kolekar et al., 2016) and used to introgress LLS and rust resistance into elite varieties (Janila et al., 2016). Also, SNPs for LLS and rust were developed and are under validation for use in molecular breeding (Pandey et al., 2017). However, to achieve the desired impact both major and minor QTLs need to be identified. Several approaches such as marker-assisted recurrent selection and GS have been proposed to identify the minor genes and improve the durable resistance. The multi-environment LLS and rust phenotyping data presented in the paper will be combined with whole genome sequencing data to develop genomic selection prediction models that can be utilized to detect the small effects QTLs (Meuwissen et al., 2001). The GS model can then be used in a breeding program to select genotypes for crossing nurseries and individuals of early generations based on their GEBV without laborious phenotypic work. GS is the best approach to capture the effect of each and every minor QTL and increases the frequency of favorable alleles in individuals of advanced generations. GS is one of the important genomic tool that can increase selection intensity and accuracy which is required to accelerate genetic gains for complex traits. Considering GEI in GEBV will be helpful to obtain the end product adaptable to a wide range of environments and can also be useful to predict the performance of genotypes in untested environments (Schulz-Streeck et al., 2013).

The significant G × E interaction effects indicate the need to identify stable sources of resistance that can perform better under a wide range of environments. The GGE biplot analysis for disease severity to LLS and rust at 90 DAS explained high proportion of variation (∼90%) due to GEI. The ranking of 109 genotypes of GSTP based on their disease severity score and stability performance identified eight genotypes stable for resistance to LLS, whereas 24 as stable for rust across the environments.

The position of environments on biplot revealed positive correlation among environments. The results indicated ICRISAT_R15 as best environment for screening for LLS and rust followed by Aliyarnagar and Jalgaon. It could be attributed to the better artificial foliar disease screening facilities available at ICRISAT. The moderate disease pressure at Jalgoan could be attributed to unfavourable environmental components such as low humidity (< 85%) and lack of rains during disease infection, establishment and spread. Environmental factors especially relative humidity, temperature, and rainfall plays an important role in disease infection and establishment of rust and LLS (Nigam et al., 1991; Cu and Phipps, 1993). For the conidial production by *Phaesaeriopsis personata*, a minimum of ≥95% relative humidity for 4 h per day is needed whereas highest numbers of conidia were produced when the lesions were subjected to ≥95% relative humidity for 16 h or more (Alderman and Nutter, 1994). Besides these, sowing at Jalgaon (23 June 2015) was nearly 15 days earlier compared to Aliyarnagar (07 July 2015) and ICRISAT (10 July 2015). Hence, the genotypes could have possibly escaped the peak disease period resulting in low infection. The significant influence of sowing time on disease severity of rust and LLS was earlier reported by Naidu and Vasanthi (1995).

The ultimate aim of the breeder is to develop genotypes which have high and stable yield performance along with disease resistance across environments/locations. In the present study, biplot analysis identified stable genotypes for pod yield that performed consistently across the environments as-well-as genotypes that are well adapted to the specific environment. Finding environment specific adaptability is also important to develop cultivars for a targeted region with region-specific adapted traits. The stable genotypes across the environments can be released after evaluation and comparison with popular national checks. Genotypes with stable yield performance were earlier reported by Mothilal et al. (2010b), Hariprasanna et al. (2008), and Pradhan et al. (2010). The biplot for pod yield per hectare shows that among the four environments, ICRISAT_PR15 plotted separately indicating that the performance of genotypes during the post-rainy season was different compared to the rainy season at ICRISAT. The superior performance of genotypes during the post-rainy season can be attributed to disease-free condition. In the present study, most of the stable genotypes for yield and its contributing traits are improved breeding lines. The genotypes from mini-core and reference set collection do not possess high yield potential but can contribute desirable genes or QTLs for other traits like disease resistance and nutritional quality traits (Upadhyaya et al., 2014 and Patil et al., 2014). Different germplasm lines with disease resistance and nutritional quality traits were earlier identified in mini-core collection (Upadhyaya et al., 2005).

## Conclusions

The present study evaluated ICRISAT’s GSTP of peanut for resistance to late leaf spot and rust. The GSTP comprising 340 genotypes including trait-specific advanced breeding lines from ICRISAT and UAS, Dharwad, lines from ICRISAT’s mini-core and reference set collection, and popular varieties cultivated in India. The study identified genotypes resistant to LLS and rust under natural and artificial diseases epiphytotic conditions. The resistant genotypes are also useful for recycling as elite parents in peanut breeding program. The hurdle of late maturity associated with resistance to LLS and rust can be overcome using early maturing sources (ICGV 86699, ICGV 01274 and SPS 8) identified in the study. Majority of the lines in GSTP were evaluated for LLS and rust for first time and the extensive variability in early and medium maturing lines indicates a positive step for improvement of LLS and rust resistance in peanut. High heritability coupled with high GAM for resistance across the environments result in greater response to selection. Understanding on mechanism of resistance in genotypes identified for specific adaptation and wide adaptation will enable the peanut breeders to diversify the genetic base of resistance to foliar fungal diseases. Significant differences in resistance among studied environments and influence of G × E interactions on resistance suggests that deployment of target ecology specific resistance to LLS and rust will be beneficial. The extensive losses of pod and haulm yield and quality caused by LLS and rust across the rainfed production environments and the pod yield increase as a consequence of resistance offered to foliar fungal diseases suggests the possibility of considering ‘foliar fungal disease resistance’ as a must-have trait in all the groundnut cultivars that will be released for cultivation in rainfed ecologies in Asia and Africa.

## Data Availability Statement

All datasets generated for this study are included in the article/**Supplementary Files**.

## Author Contributions

JP and SC designed the experiment; SC carried out experiment at ICRISAT, Patancheru, collected, analyzed data, prepared tables, interpreted the results, and written manuscript. JP constituted the GSTP based on the available historical data; DK and JP has prepared work plan and reviewed the manuscript critically, SP and SS conducted trials and recorded data at Jalgaon and Aliyarnagar, respectively. MV helped in drafting and revising the manuscript critically, HS prepared disease screening nursery and inoculum of spores for both the fungus. SM helped in generating field layout, multiplying and sending seeds, and technical resources during the course of experiment. RB has shared some of the genotypes for the study. All authors have read and approved the final manuscript.

## Funding

The authors are thankful to CRP-Grain Legumes and Dryland Cereals (CRP-GLDC) for financing the research work and Bill and Melinda Gates Foundation for providing the scholarship to the first author and financial support to conduct this experiment.

## Conflict of Interest

The authors declare that the research was conducted in the absence of any commercial or financial relationships that could be construed as a potential conflict of interest.
