# Supplementary material for: Genotype × Environment Studies on Resistance to Late Leaf Spot and Rust in Genomic Selection Training Population of Peanut (Arachis hypogaea L.)
Source: Front Plant Sci. 2019 Dec 4;10:1338. doi: 10.3389/fpls.2019.01338 (PMC6904303; doi:10.3389/fpls.2019.01338)
Supplement: Supplementary file 3 [file Table_3.docx]

**Supplementary Table 3 Categorization of genotypes of GSTP of peanut against LLS based on field evaluation across the three locations during rainy 2015**

| **Reaction*** | **Number** | **Genotypes** |
| --- | --- | --- |
| Resistant | 31 | 49 M- 1-1, 49 M-16, GPBD 4, ICG 11337 , ICG 6022, ICGVs 00068, 00246, 00248, 00362, 01274, 01328, 02323, 02446, 02411, 03043, 04087, 05036, 05100, 05141, 05163, 06142, 07235, 86699, 99051, 99052, 99160, M 28-2, SPS 11, SPS 2, SPS 20 and SPS 8 |
| Moderately Resistant | 162 | 24 × 39-31 MR, 24 M-86, 26 × 37-IV- 9IR, 26 × M-223-1, 26 × M-95-1 RI, 26 M 156-2, 27 × 49- 12, 27 × 49- 14, 27 × 49- 16, 39 × 49 -77, 39 × 49 -8, 39 × 49-81-1, 49 × 37-134, 49 × 27-37, 49 × 37-90, 49 × 39-21-1, 49 × 39-21-2, 49 × 39-21-2(a), 49 × 39-74, 49 × 39-8, 49 M-2-2, BAU 13, CS 39, CSMG 84-1, DH 86, Gangapuri, ICGs 10036, 10053, 10185, 11322, 11426, 12276, 12370, 12509, 12625, 13895, 14466, 14475, 14482, 14705, 14834, 15415, 15419, 156 (M 13), 2381, 2773, 2857, 4527, 4343, 5221, 532, 5662, 5745, 5891, 6646, 721, 6766, 8285, 875, 8751, 9961, ICGS 76, ICGVs 00005, 00191, 00290, 00346, 00350, 00351, 00371, 01060, 01124, 01265, 01273, 01276, 01361, 01393, 01464, 01495, 02189, 02242, 02286, 02287, 02290, 02317, 02321, 02434, 03042, 03056, 03064, 03128, 03136, 03397, 04044, 04115, 05032, 05057, 05155, 05161, 06040, 06042, 06099, 06100, 06110, 06175, 06188, 06422, 06423, 06420, 06424, 07120, 07145, 07148, 07166, 07168, 07220, 07223, 07227, 07246, 07247, 07368, 86325, 86590 , 87160, 87187, 87846, 90320, 93216, 93920, 93280, 94118, 95058, 97045, 97058, 97092, 97115, 97116, 97120, 97128, 97165, 97182, 98105, 98163, 98184, 98373, 99029, 98432, 99085, ICR 48, M 110-14, M 28-2, MN1-35, Somnath, SPS 1, SPS 14, SPS 15, SPS 21, SPS 7, SPS 9, TDG 13, TDG 14, TKG 19A and TPG 41 |
| Susceptible | 147 | 24 × 37-2275, 26 × 27-164, 26 M- 119-1, 27 × 49- 27-1, 49 × 27-19, 49 × 37- 99(b) tall, 49 × 37-135, 49 × 37-97-1, DTG 3, Faizpur 1-5, ICGs 10701, 11088, 11651, 111, 12672, 14985, 15190, 1668, 1834, 2106, 3027, 3053, 3102, 3140, 3421, 3746, 434, 4955, 5663, 8517, ICGS 11, ICGS 44, ICGVs 00343, 00387, 00440, 01005, 01263, 01478, 02022, 02125, 02144, 02206, 02251, 02266, 02271, 02298, 03184, 03398, 03207, 04018, 04124, 04149, 05176, 05198, 06049, 06347, 06234, 06431, 07023, 07210, 07268, 07359, 09112, 13238, 13241, 13245, 86011, 86015, 86072, 86143, 86352, 86564, 87354, 87378, 87921, 88145, 88438, 89104, 91114, 92195, 92267, 93437, 94169, 94361, 95070, 95290, 95377, 95469, 96468, 97183, 97232, 97261, 98294, 99083, 99195, 99233, JL 24, Mutant 3, SPS 10 , SPS 13, SPS 17, SPS 3, SPS 6, Sun Oleic 95R, TDG 10, TG 39, TG 41, TG 19, TG 42, TG 49, TG LPS 3, TG LPS 4, TG LPS 7, TMV 2 NLM, 49 × 27-13 (ii), 49 × 37-91, 49 × 39-20-2, DTG 15, ICGs 12879, ICGs 12991, 1973, 2031, 3312, 3343, 3584, 3673, 4543, 442, 4729, 9315, 9507, ICGVs 00321, 00349, 01232, 02038, 02194, 07217, 07273, 13242, 91116, 93470, 96466, 97262, 99181, J 11, TAG 24 and TMV 2 |

* Genotypes were categorized based on Resistant/susceptible reaction to LLS disease on a 1-9 scale where Resistant (R) =1-3; Moderately Resistant (MR) = 4-5; Susceptible (S) = 6-7; Highly susceptible (HS) = 8-9 disease severity rating scale
