# Supplementary material for: Genotype × Environment Studies on Resistance to Late Leaf Spot and Rust in Genomic Selection Training Population of Peanut (Arachis hypogaea L.)
Source: Front Plant Sci. 2019 Dec 4;10:1338. doi: 10.3389/fpls.2019.01338 (PMC6904303; doi:10.3389/fpls.2019.01338)
Supplement: Supplementary file 4 [file Table_4.docx]

**Supplementary Table 4 Categorization of genotypes of GSTP of peanut against rust based on field evaluation across the three locations during rainy 2015**

| **Reaction*** | **Number** | **Genotypes** |
| --- | --- | --- |
| Resistant | 66 | 39 × 49-81-1, 49 × 39-74, 49 M- 1-1, 49 M-16,CS 39, GPBD 4, ICG 11337, ICG 11426, ICG 8751, ICGVs 00005, 00068, 00191, 00248, 00246, 00346, 00362, 01265, 01273, 01274, 01276, 01361, 01464, 02317, 02323, 02411, 02446, 03042, 03043, 03064, 04087, 05032, 05036, 05057, 05100, 05141, 05155, 05163, 06142, 06175, 06422, 06423, 06424, 07120, 07145, 07220, 07223, 07227, 07247, 07235, 86590 , 86699, 87846, 97128, 98105, 98373, 99051, 99029, 99052, 99085, 99160, SPS 11, SPS 2, SPS 20, SPS 21, SPS 7 and SPS 8 |
| Moderately Resistant | 138 | 24 × 37-2275,24 × 39-31 MR,24 M-86,26 × M-223-1, 26 × M-95-1 RI, 27 × 49- 12, 27 × 49- 14, 39 × 49 -77, 39 × 49 -8, 49 × 27-37, 49 × 37-134, 49 × 37-90, 49 × 39-21-2, 49 × 39-21-1, 49 × 39-21-2(a), 49 × 39-8, 49 M-2-2, BAU 13, CSMG 84-1, DH 86, ICGs 10036, 10053, 11088, 111, 12276, 12370, 12509, 12625, 13895, 14466, 14475, 14482, 14834, 15190, 15415, 15419, 2381, 2773, 2857, 3027, 3053, 4343, 4527, 532, 5662, 5745, 6022, 6766, 6646, 8285, 875, ICGS 76, ICGVs 00290, 00350, 00351, 00440, 00387, 01060, 01124, 01263, 01328, 01393, 01478, 01495, 02194, 02206, 02242, 02266, 02286, 02287, 02290, 02321, 02434, 03056, 03128, 03136, 03207, 03397, 03398, 04044, 04115, 04124, 05161, 05176, 05198, 06040, 06042, 06099, 06100, 06188, 06420, 07148, 07166, 07168, 07210, 07246, 07368, 13241, 86325, 86564, 87187, 87921, 88438, 90320, 93216, 93920, 94118, 93280, 95469, 96466, 97045, 97058, 97092, 97115, 97116, 97120, 97165, 97182, 98163, 98184, 98294, 98432, 99083, 99195, 99233, 01274, M 110-14, M 28-2, MN1-35, M 28-2, SPS 1, SPS 14, SPS 15, SPS 17, SPS 9, TDG 13, TDG 14, TG 42 and TG LPS 4 |
| Susceptible | 136 | 26 × 27-164, 26 × 37-IV- 9IR, 26 M- 119-1, 26 M 156-2, 27 × 49- 16, 27 × 49- 27-1, 49 × 27-13 (ii), 49 × 27-19, 49 × 37-97-1, 49 × 39-20-2, DTG 15, DTG 3, Gangapuri, ICGs 10185, 11322, 11651, 12672, 12879, 12991, 14705, 156 (M 13), 1668, 2031, 2106, 3102, 3140, 3312, 3343, 3673, 3746, 4543, 4955, 5221, 5663, 5891, 721, 8517, 9507, 9961, ICGS 11, ICGS 44, ICGVs 00321, 00343, 00349, 00371, 01232, 02022, 02125, 02038, 02144, 02189, 02251, 02271, 02298, 03184, 06049, 04018, 06234, 06347, 07023, 07217, 07268, 07273, 07359, 09112, 13238, 13242, 13245, 86011, 86015, 86072, 86143, 86352, 87160, 87354, 87378, 88145, 89104, 91116, 92195, 92267, 93437, 94169, 94361, 95058, 95290, 95377, 97183, 97232, 97261, 97262, 01005, 04149, 06110, 06431, 91114, 93470, 95070, 96468, J 11, 99181, ICR 48, JL 24, Somnath, SPS 13, SPS 3, SPS 6, Sun Oleic 95R, TAG 24, TG 39, TG 49, TG LPS 3, TKG 19A, TMV 2 NLM, TPG 41, 49 × 37-135, 49 × 37-91, 49 × 37- 99(b) tall, Faizpur 1-5, ICGs 10701, 14985, 1834, 1973, 3421, 3584, 434, 442, 4729, 9315, Mutant 3, SPS 10 , TDG 10, TG 19, TG 41, TG LPS 7 and TMV 2 |

* Genotypes were categorized based on Resistant/susceptible reaction to rust disease on a 1-9 scale where Resistant (R) =1-3; Moderately Resistant (MR) = 4-5; Susceptible (S) = 6-7; Highly susceptible (HS) = 8-9 disease severity rating scale
